# Supplementary material for: Effects of injectable contraception with depot medroxyprogesterone acetate or norethisterone enanthate on estradiol levels and menstrual, psychological and behavioral measures relevant to HIV risk: The WHICH randomized trial
Source: PLoS One. 2024 Mar 26;19(3):e0295764. doi: 10.1371/journal.pone.0295764 (PMC10965066; doi:10.1371/journal.pone.0295764)
Supplement: S1 Table — (DOCX) [file pone.0295764.s002.docx]

**S1 Table. Daily diary questionnaire**

| Please tell us what happened from mid-day yesterday until mid-day today? | | | | | | | | | | | | | | | | | | | |  |  |  |  |  | |  | |  |  |
| --- | --- | --- | --- | --- | --- | --- | --- | --- | --- | --- | --- | --- | --- | --- | --- | --- | --- | --- | --- | --- | --- | --- | --- | --- | --- | --- | --- | --- | --- |
|  |  |  |  |  |  |  |  |  |  |  |  |  |  |  |  |  |  |  |  |  |  |  |  |  | |  | |  | |
|  |  |  |  |  |  |  |  |  |  |  |  |  |  |  | **1** | **2** | **3** | **4** | **5** | **6** | **7** | **8** | **……..** | | **28** | |  |  |  |
| **1** | **Have you been menstruating?** | | | | | | | |  |  |  |  |  |  |  |  |  |  |  |  |  |  |  | |  | |  |  |  |
|  | 0=No |  | 2.Light | | |  |  |  |  |  |  |  |  | **1** |  |  |  |  |  |  |  |  |  |  |  |  |  |  |  |
|  | 1.Normal |  | 3.Heavy | | |  |  |  |  |  |  |  |  |  |  |  |  |  |  |  |  |  |  |  |  |  |  |  |  |
| **2** | **Has your menstruation been?** | | | | | | | |  |  |  |  |  |  |  |  |  |  |  |  |  |  |  | |  | |  |  |  |
|  | 0.No menstruation | | | |  | 2.Mild pain | | | |  |  |  |  | **2** |  |  |  |  |  |  |  |  |  |  |  |  |  |  |  |
|  | 1.Painless menstruation | | | | | 3.Severe pain | | | | |  |  |  |  |  |  |  |  |  |  |  |  |  |  |  |  |  |  |  |
| **3** | **Have you had sexual intercourse? If yes was it?** | | | | | | | | | |  |  |  |  |  |  |  |  |  |  |  |  |  | |  | |  |  |  |
|  | 0.No intercourse | | |  | | 2.Casual partner | | | | | |  |  | **3** |  |  |  |  |  |  |  |  |  |  |  |  |  |  |  |
|  | 1.Steady partner only | | | | |  |  |  |  |  |  |  |  |  |  |  |  |  |  |  |  |  |  |  |  |  |  |  |  |
| **4** | **If yes Q4 sexual intercourse? Was condom used?** | | | | | | | | | | | | | |  |  |  |  |  |  |  |  |  |  |  |  |  |  |  |
|  | 0.No intercourse | | |  |  | 2.No condom | | | | |  |  |  |  |  |  |  |  |  |  |  |  |  | |  | |  |  |  |
|  | 1.Yes condom | | |  |  |  |  |  |  |  |  |  |  | **4** |  |  |  |  |  |  |  |  |  |  |  |  |  |  |  |
| **5** | **Have you felt sad for no real reason?** | | | | | | | | | | |  |  |  |  |  |  |  |  |  |  |  |  |  |  |  |  |  |  |
|  | 0 = | No | |  |  |  |  |  |  |  |  |  |  |  |  |  |  |  |  |  |  |  |  | |  | |  |  |  |
|  | 1 = | Yes | |  |  |  |  |  |  |  |  |  |  | **5** |  |  |  |  |  |  |  |  |  |  |  |  |  |  |  |
| **6** | **Have you felt an urge to have sexual intercourse?** | | | | | | | | | | | | | |  |  |  |  |  |  |  |  |  |  |  |  |  |  |  |
|  | 0 = | No | |  |  |  |  |  |  |  |  |  |  |  |  |  |  |  |  |  |  |  |  | |  | |  |  |  |
|  | 1 = | Yes | |  |  |  |  |  |  |  |  |  |  | **6** |  |  |  |  |  |  |  |  |  |  |  |  |  |  |  |
| **7** | **Did you feel that your partner loves you?** | | | | | | | | | | | |  |  |  |  |  |  |  |  |  |  |  |  |  |  |  |  |  |
|  | 0 .No |  |  |  | 2 = | No partner | | | |  |  |  |  |  |  |  |  |  |  |  |  |  |  | |  | |  |  |  |
|  | 1 .Yes |  |  |  |  |  |  |  |  |  |  |  |  | **7** |  |  |  |  |  |  |  |  |  | |  | |  |  |  |
