# Supplementary material for: Effects of injectable contraception with depot medroxyprogesterone acetate or norethisterone enanthate on estradiol levels and menstrual, psychological and behavioral measures relevant to HIV risk: The WHICH randomized trial
Source: PLoS One. 2024 Mar 26;19(3):e0295764. doi: 10.1371/journal.pone.0295764 (PMC10965066; doi:10.1371/journal.pone.0295764)
Supplement: S2 Table — (DOCX) [file pone.0295764.s003.docx]

**S2 Table. Spearman correlations between estradiol (pmol/L) levels and BDI, ASEX and**

**Daily Diary scores**

|  | **n** | **Correlation** | **p-value** |
| --- | --- | --- | --- |
| **Estradiol and BDI** | | | |
| Baseline | 514 | -0.10 | 0.026 |
| 25 weeks | 447 | -0.14 | 0.004 |
| **Estradiol and ASEX** | | | |
| Baseline | 514 | 0.09 | 0.054 |
| 25 weeks | 447 | 0.11 | 0.018 |
| **25 week estradiol and number of sexual acts per 28 days** | 418 | -0.07 | 0.161 |
| **25 week estradiol and number of condomless sexual acts per 28 days** | 418 | -0.01 | 0.788 |
| **25 week estradiol and number of days with urge for sexual intercourse** | 418 | 0.04 | 0.467 |
