# Supplementary material for: Effects of injectable contraception with depot medroxyprogesterone acetate or norethisterone enanthate on estradiol levels and menstrual, psychological and behavioral measures relevant to HIV risk: The WHICH randomized trial
Source: PLoS One. 2024 Mar 26;19(3):e0295764. doi: 10.1371/journal.pone.0295764 (PMC10965066; doi:10.1371/journal.pone.0295764)
Supplement: S1 File — (PDF) [file pone.0295764.s004.pdf]

Study title: *The Women's Health, Injectable Contraception and HIV study (Part 1): randomized comparison of immunological, hormonal, physiological, psychological and behavioural effects of NET-EN versus DMPA contraception.*

Protocol

Version 0.1

Funder :

South African Medical Research Council.

GENERAL INFORMATION

Study title: The Women's Health, Injectable Contraception and HIV study (Part 1): randomized comparison of immunological, hormonal, physiological, psychological and behavioural effects of NET-EN versus DMPA contraception.

Short Title: The WHICH injection study (Part 1)

Trial registration number:

Sponsor: South African Medical Research Council

Wits Ethics Clearance Certificate no:

Protocol versions:

Protocol version 1.0	31 July 2018

COLLABORATORS

Overall study Investigator	Dr Mandisa Singata-Madliki (PhD)<br>Effective Care Research Unit (ECRU)*, University of Fort Hare	
Co-Principal Investigators	1. Professor Jennifer Smit (PhD)<br>Match Research Unit<br>University of Witwatersrand	2. Prof Janet Patricia Hapgood<br>Molecular and Cell Biology Building<br>University of Cape Town
Site Co-Investigators	1.Dr Joanne Batting<br>Effective Care Research Unit (ECRU),<br>University of Fort Hare<br><br>2.Dr Mags Beksinska<br>Match Research Unit<br>University of Witwatersrand	3.Dr Chanel Avenant<br>Molecular and Cell Biology Building<br>University of Cape Town
Advisors	1.Prof G. Justus Hofmeyr<br>Effective Care Research Unit (ECRU),<br>University of Witwatersrand/University of Fort Hare, South Africa<br><br>2.Prof Marleen Temmerman<br>Chair, Department OB/GYN<br>Director, Centre of Excellence in Women and Child Health ,Aga Khan University<br>Parklands Avenue,00100 Nairobi, Kenya	3. Dr Charles Morrison, PhD<br>Senior Scientist<br>FHI 360<br>Durham, NC

ABBREVIATIONS AND DEFINITIONS OF TERMS

DMPA	Depot medroxyprogesterone acetate
NET-EN	Norethisterone enanthate
AE	Adverse Event
CONSORT	Consolidated Standards Of Reporting Trials
CRF	Case Report Forms
DSMC	Data Safety and Monitoring Committee
ECRU	Effective Care Research Unit
GCP	Good Clinical Practice
ICF	Informed Consent Form
ID	Identification
PI	Principal Investigator
SAE	Serious Adverse Event
TSC	Trial Steering Committee
WHO	World Health Organization
BDI-II	Beck Depression Inventory
MADRS	Montgomery-Asberg Depression Rating Scale
ASEX	Arizona Sexual Experience Scale
SAMRC	South African Medical Research Council
IQR	Interquartile range
MEC	Medical eligibility criteria for contraceptive use
STI	Sexually transmitted infection
PCR	polymerase chain reaction
RCT	Randomised Clinical Trail
SAHPRA	South African Health Products Regulatory Authority
IP	Injectable Progestogens

TABLE OF CONTENTS

GENERAL INFORMATION 2
COLLABORATORS 3
ABBREVIATIONS AND DEFINITIONS OF TERMS 4
TABLE OF CONTENTS 5
PROJECT SUMMARY 7
1. Background information and Rationale 9
1.1. Justification of choice of study arms 13
1.1.1 DMPA 13
1.1.2. NET-EN 13
2. Literature review: hormonal contraception and HIV 14
2.1. Review of observational data on HIV acquisition and progestin-only contraceptives 14
2.2. Review of biological data on HIV acquisition and progestin-only contraceptives 14
2.3.Justification for a randomized, controlled trial 15
2.4. Feasibility of a randomized, controlled trial 15
2.5. Feasibility of offering each of the two study methods in selected study sites 15
3. Study objectives 15
4. Study outcomes: 16
4.1 Primary outcomes 16
4.2 Secondary outcomes 16
5. Study design 16
5.1 Methods and study procedures : 17
5.2 Study sites 17
5.3 Study participants 17
5.3.1 Participant recruitment 17
5.4 Eligibility Criteria 18
5.4.1 Inclusion criteria 18
5.4.2 Exclusion criteria 18
5.5 Sampling and allocation 18
5.6 Sample size calculation 19
5.7 Description of the intervention 19
5.7.1 Drugs and devices 19
5.7.2 Administration of study products 20
5.8 Study procedures 20
5.8.1 Recruitment and screening 20
5.8.2 Enrolment procedures 20

5.8.3 Randomization procedure 22
5.8.4 Follow-up visits 22
5.8.5 Study Follow-up procedures 23
5.8.6 Contraceptive follow-up after final research visit 25
5.9 Criteria for discontinuation of a participant 25
5.10 Criteria for discontinuation of the study 25
6. Study instruments 25
6.1 Questionnaire 25
6.2 Beck Depression Inventory (BDI-II) 25
6.3 Arizona Sexual Experience Scale 26
6.4 28 Days daily Dairy 26
7. Project management 26
7.1 Data quality assurance 26
8. Data management 27
8.1 Confidentiality of data 27
8.2 Data analysis plan 27
8.3 Study timeline 28
9. Main problems anticipated and proposed solutions 28
10.Applicability of results 29
11. Ethical considerations: 29
12.1. Study population, recruitment strategy and informed consent process 29
12.2 Perceived risks and benefits of the study, both at the individual and community levels .. 29
12.3 Safeguards to protect any recognized vulnerability of the study participants 30
12.4 Reimbursement or compensation to study participants 30
12.5 Access to treatment or counselling for conditions either identified during screening of potential participants or resulting from the study intervention 30
12.6 Responsiveness of the project to community needs and priorities 31
12.7 Deception 31
13. Gender considerations 31
13.1 Describe how women and men are affected by the public health need that the study addresses, and whether this is a need expressed or felt by women and/or men 31
13.2 Explain how the research contributes to identifying and/or reducing inequities between women and men in sexual and reproductive health and health care. 31
13.3 Describe measures taken to facilitate the individual participation of women or men in the research process in light of their different life situations 31
13.4 Describe measures taken to ensure that community involvement is inclusive 31

14. Environmental impact of the project ... 31
15. Plans for dissemination and use of project results ... 32
16. Budget ... 32
Appendix 1: 28 Days Daily Diary ... 33
17 .References ... 34

PROJECT SUMMARY

Title: The Women's Health, Injectable Contraception and HIV study (Part 1): randomized comparison of immunological, hormonal, physiological, psychological and behavioural effects of NET-EN versus DMPA contraception.

Short Title: WHICH INJECTION (PART 1)

Objective

1. To measure differences in immunological, hormonal, physiological, psychological, behavioural and hormonal effects of NET-EN versus DMPA considered to be relevant to HIV acquisition risk and method adherence.
2. To demonstrate feasibility of a proposed large randomized clinical trial to determine the relative risk of HIV acquisition.

Background

Effective contraception is the single most important strategy for reducing maternal and perinatal mortality globally. In countries such as South Africa, depot medroxyprogesterone acetate (DMPA) accounts for the great majority of modern contraceptive usage.

Animal, immunological, endocrinological and observational clinical studies have suggested that DMPA may increase susceptibility to HIV. However, the net effects of biological, physiological and behavioural effects is unknown, nor are the relative effects of alternative contraception methods.

Study design: Randomized clinical trial

Participants: Women attending health services in the collaborating Department of Health participating services, and women attending the research sites who, after routine counselling on all the methods available (Injectables, OC, IUD, Implant), request and are assessed as suitable for injectable contraception and are HIV negative, will be eligible for participation.

Interventions:

1. Norethisterone enanthate 200mg intramuscular (NET-EN)
2. Depot medroxyprogesterone acetate 150mg intramuscular (DMPA IM)

Primary outcomes:

The primary hormonal outcome will be serum 17β estradiol, and the primary psychological outcome will be depression (BDI-II). The study will be powered to measure a difference of 35 pmol/L in the mean serum 17β estradiol between arms, and a difference of 2 in the mean depression scores (BDI-II).The other measures will be regarded as exploratory.

Other Outcomes:

- Sentinal immune suppression markers (eg CCR5 levels on T cells and CD14+ cells from genital tract samples; plasma interferon γ).
- Serum sex hormone binding globulin
- Serum testosterone
- Serum free testosterone
- Serum progesterone
- Additional depression measures
- Sexual dysfunction

- Sexual activity including condom use
- Amenorrhoea
- Weight change
- Adherence

Methods

HIV negative women between the age of 18-40years attending family planning services or the research sites will be fully counselled on all available contraceptive options.Those who request IP and meet the eligibility criteria will be informed of the trial and offered participation. Baseline demographic, psychological, physiological and behavioural data will be recorded. Baseline blood and genital tract samples for immunological and hormonal studies will be collected. Participants will be allocated by an online randomization service to one of the 2 IP options, and receive their first injection. A 28-day daily symptom and behaviour diary will be initiated at month 6 to be returned at month 7, 8 or 9. if funds are permit we will use an sms system for particpants to complete the daily diary. Blood will be collected for immunological and hormone studies at baseline month 0, month 6 and at month 6+7days. Subject to funding, HIV PCR will be measured at 18 months.

Sample size was calculated based on the expected standard deviation of key continuous outcomes, (221 participants per group), and allowing for 15% loss to follow-up, will be 260 per group.

Analysis

Analysis will be by intention to treat, and the results will be reported according to the CONSORT guidelines. Appropriate transformation of non-normal or skewed measurement will be performed on continuous variables. If transformations do not generate a distribution approaching normality, medians and IQR will be reported. For incidence outcomes (eg pregnancy and HIV seroconversion) with varying duration of follow up between groups, data will be adjusted for duration of follow up.
Categorical variables will be compared as risk ratios with 95% confidence intervals. For continuous data, mean differences with 95% confidence intervals will be reported.
Value: The need for generating definitive evidence on the relative safety or risks of NET-EN versus DMPA is urgent. A randomized study of comparative effects related to HIV risk will provide the best available evidence to date quickly and efficiently.

1. Background information and Rationale

Effective contraception is the single most important strategy for reducing maternal and perinatal mortality globally. In countries such as South Africa, depot medroxyprogesterone

acetate (DMPA) accounts for the great majority of modern contraceptive usage. Animal, immunological, endocrinological and observational clinical studies have suggested that DMPA may increase susceptibility to HIV. However, the net effect of biological, physiological and behavioural effects is unknown, nor are the relative effects of alternative contraception methods. The international collaborative ECHO study currently being conducted will address the relative effects of DMPA versus the intrauterine device (IUD) and the levonorgestrel (LNG) implant. However, our experience over more than 10 years of providing the IUD and more recently the etonorgestrel implant has indicated that the majority of women in the Eastern Cape and elsewhere in South Africa continue to prefer injectable contraception. Reasons include familiarity as this has been the dominant method in these communities for decades, confidentiality and fear of complications with the IUD and implant. Thus, the most important question in terms of potential public health benefit and implementation, is the relative effects of alternative injectables, norethisterone enanthate (NET-EN) versus DMPA IM.

The primary objective of the proposed study (part 1) is to provide robust evidence to inform clinicians, policy-makers, and women about the relative risks and benefits of NET-EN versus DMPA IM with respect to biological, physiological, hormonal, psychological and behavioural effects, and method continuation. This question is relevant for all settings where women prefer injectable contraception.

HIV acquisition

The WHO Guidance statement on hormonal contraceptive eligibility for women at high risk of HIV (2 March 2017)

The WHO re-examined this issue in Dec 2016. Relevant excerpts from their published guidance follow: "The World Health Organization (WHO) convened a technical consultation during 1–2 December 2016 to review new evidence on the risk of HIV acquisition with the use of hormonal contraception. The issue was recognized as a critical one, particularly for sub-Saharan Africa, where women have a high lifetime risk of acquiring HIV, hormonal contraceptives constitute a significant component of the contraceptive method mix and unintended pregnancy is a common threat to the well-being and lives of women and girls.

"Women at high risk of acquiring HIV can also use progestogen-only injectables (norethisterone enanthate [NET-EN] and depot medroxyprogesterone acetate [DMPA, intramuscular or subcutaneous] because the advantages of these methods generally outweigh the possible increased risk of HIV acquisition (MEC category 2).

"There continues to be evidence of a possible increased risk of acquiring HIV among progestogen-only injectable users. Uncertainty exists about whether this is due to

methodological issues with the evidence or a real biological effect. In many settings, unintended pregnancies and/or pregnancy-related morbidity and mortality are common, and progestogen-only injectables are among the few types of methods widely available. Women should not be denied the use of progestogen-only injectables because of concerns about the possible increased risk. Women considering progestogen-only injectables should be advised about these concerns, about the uncertainty over whether there is a causal relationship, and about how to minimize their risk of acquiring HIV.
"There is… insufficient evidence to determine if the HIV acquisition risk associated with NET-EN differs from the risk associated with DMPA…

"One challenge when formulating recommendations for contraceptive use among women at high risk of HIV infection has been the absence of evidence from randomized clinical trials on the topic.
"The 'Evidence for Contraceptive Options and HIV Outcomes' (ECHO) trial is an ongoing randomized trial that seeks to provide definitive information on the risk of HIV acquisition associated with different contraceptive methods…. DMPA, an LNG implant or a copper IUD. …Study results will not be available before 2019."

The importance of addressing this question is further highlighted by the circumstance that many of the countries most affected by the HIV epidemic are also those where women have the fewest contraceptive options available to them, and many rely on DMPA1. Further complicating matters are the competing risks women must weigh in settings where forgoing family planning puts them at significant risk of maternal morbidity or mortality2.
If the ECHO trial finds higher risk of HIV acquisition among women randomized to DMPA than to either of the comparators without robust evidence available on the potential alternative injectable NET-EN, the negative impact on family planning programmes may result in increased maternal and perinatal mortality from unintended pregnancies. Moreover, the increase in unintended pregnancies may increase women's risk of HIV due to the progestogenic immunosuppressive effects of pregnancy. Observational evidence suggests that HIV risk increases to about 2.8-fold in late pregnancy and 4-fold postpartum3 . Another observational study in South Africa found that pregnant women reported more sexual partners and condomless sex than postpartum women4.

Mechanistic hypotheses have focussed on mechanisms whereby DMPA might increase HIV susceptibility, but the opposite is also feasible. On the one hand, DMPA may increase risk due to immunological suppression and hypo-estrogenism. On the other hand, DMPA may reduce

risk by behavioural changes related to reduced libido 5 , increased condom use and amenorrhoea, reducing sexual exposure during menstruation6.

Contraceptive options and unintended pregnancy in South Africa

Women in South African health services currently have limited contraceptive choices. In most cases, only oral contraceptive pills and injectable contraceptives (and more recently the etonorgestrel implant) are offered. Unfortunately, injectables have a high discontinuation rate (nearly 50% will discontinue use in 12 months)7, largely due to menstrual disturbances. The result of this is high rates of unintended pregnancy. In a survey of post-partum women in our East London service in South Africa, two-thirds reported that their most recent birth was unplanned. Although 79% of the women had previously used contraception, many had discontinued, 32% of whom did so due to side effects.

Progestin-only injectable (DMPA and NET-EN)

Injectable progestogen contraceptives are acceptable to many women, and have the benefits of convenience, infrequent dosage (every three months for DMPA), confidentiality, a high efficacy rate when used correctly, and their safety for most women, even those with medical conditions or who are post-partum or breastfeeding (and at least six weeks post-partum). However, they have some features that are less desirable for some women, which may lead to discontinuation and unintended pregnancies. The most common side effects include irregular vaginal bleeding, amenorrhea, and weight gain. Additionally, return to fertility can take up to nine months, and the need to attend the health service every two to three months for injections can be inconvenient for women.

Use of progestin-only contraceptives post-partum

WHO's Medical Eligibility Criteria has no restrictions on the use of progestin-only contraceptives (POCs) immediately post-partum in non-breastfeeding women. However, their use is classified category 3 in the first 6 weeks post-partum for women who are breastfeeding due to theoretical concerns of the risk of exposure of the neonate to steroid hormones; at 6 weeks and beyond, progestin-only contraceptives are classified as category 2. It is noted that in some settings where access to services is limited and morbidity and mortality from pregnancy is high, POCs may be one of the few types of methods widely available and accessible to breastfeeding, post-partum women. South Africa's Contraceptive Clinical Guidelines note the following:

"It is recommended that, ideally, initiation of progestogen-only injectables is delayed until six weeks postpartum. However, this puts women who do not fully breastfeed or discontinue breastfeeding before six weeks at risk of early conception (ovulation can start as early as 28

days postpartum). Thus, if no other methods are available or acceptable to the client, progestogen injectables could be initiated prior to discharge from the health facility"8.

Breastfeeding rates in South Africa are low, with only 25% of women exclusively breastfeeding9. In light of this, the limited availability of alternative contraceptive methods and the preference of many women for their use, progestin-only injectables are in practice routinely provided in the immediate post-partum period in the South African setting.

Norethisterone Enanthate (NET-EN)

Limited evidence from observation studies suggests a lower association of HIV acquisition with NET-EN than DMPA[10]. Again, there is no evidence of causality. Differences may be due to differences in the characteristics of women who receive NET-EN. For example, health providers tend to recommend NET to younger women and to women they perceive to be 'reliable', because of quicker return to fertility,.

1.1. Justification of choice of study arms

1.1.1 DMPA

DMPA (150 mg of medroxyprogesterone acetate (MPA)/ml) administered IM every 12 weeks) is the most commonly used injectable contraceptive worldwide, and the most commonly used method of reversible contraception in South Africa.

1.1.2. NET-EN

NET-EN (norethisterone enanthate 200mg/ml administered IM every 8 weeks) is another common progestin-only injectable contraceptive method used in developing countries and is especially popular among young women in South Africa.

We have chosen to study the effects of DMPA for the following reasons :

Previous animal, immunological and observational clinical research has shown that among available contraceptive methods, DMPA is theoretically the most likely hormonal contraceptive to increase HIV acquisition in women.

DMPA is very widely used in South Africa, which has the largest HIV epidemic in the world, as well as in other sub-Saharan African countries.

Very limited biological and epidemiological data suggest that the risk with NET-EN may be lower than with DMPA

For these reasons, the need for generating definitive evidence on the relative safety or risks of NET-EN versus DMPA is urgent. A randomized study of comparative effects related to HIV risk will provide the best available evidence to date quickly and efficiently.

2. Literature review: hormonal contraception and HIV

2.1. Review of observational data on HIV acquisition and progestin-only contraceptives

A systematic review conducted to evaluate the impact of hormonal contraception on HIV acquisition among HIV-negative women found that the preponderance of data for oral contraceptive pills, injectable norethisterone enanthate, and levonorgestrel implants do not suggest an association with HIV acquisition, though data for implants are limited. The new, higher quality studies on DMPA (or nondisaggregated injectables), which had mixed results in terms of statistical significance, had hazard ratios between 1.2 and 1.7, consistent with the meta-analytic estimate for all higher quality studies of hazard ratio 1.4.

The authors concluded that although confounding in these observational data cannot be excluded, new information increases concerns about DMPA and HIV acquisition risk in women. If the association is causal, the magnitude of effect is likely to be a hazard ratio 1.5 or less. Data for other hormonal contraceptive methods, including norethisterone enanthate, are largely reassuring but too limited to be considered definitive.

2.2. Review of biological data on HIV acquisition and progestin-only contraceptives

Multiple biological mechanisms exist that could explain an effect of hormonal contraception on HIV acquisition, including: thinning of the vaginal epithelium with medroxyprogesterone11, although this effect is less clear in human studies than in simian models, with contradictory findings on the effect of medroxyprogesterone acetate on the vaginal epithelium12,13,14, increased frequency of Langerhans cells in the vaginal epithelium in some studies15 but not others16; increased expression and prevention of downregulation of the CCR5 receptor on cervical lymphocytes in ex vivo studies of medroxyprogesterone acetate and during physiological stages associated with high progesterone levels, such as pregnancy17; inhibition of cell-mediated immune response in ex vivo studies of human lymphocytes with medroxyprogesterone acetate,18,19 and in macaque studies20, as well as in studies comparing plasma and cervicovaginal cytokine levels of women using DMPA with those using oral contraceptives or no hormonal contraceptive. Studies have shown that some of these effects are evident when cells are incubated with medroxyprogesterone acetate, but not with unmodified progesterone or estrogen, or with NET-EN21, suggesting that DMPA may have effects that are different from other hormonal contraceptives, be they progestin-only or combined hormonal contraceptives that also contain estrogen. Mouse and human studies have found that both DMPA and levonorgestrel reduce the genital mucosal barrier to infection22

While most studies have used animal models, in vitro cell cultures and vaginal biopsies, in a non-randomized human study reduced plasma interferon α in women using DMPA has been

regarded as a marker for the immunological effects which might link DMPA to increased HIV risk[18].

2.3.Justification for a randomized, controlled trial

Random allocation is the only known method of controlling for both known and unknown confounding variables. Details of the randomization method are given below. Observational data on this topic thus far has proven difficult to interpret due to the high potential for unmeasured confounding from multiple sources, including provider bias in contraceptive provision, characteristics of contraceptive users that influence both their choice of method and their risk of HIV acquisition, and difficulty measuring important confounding factors such as condom use and behavioural risk. These confounders will be minimized with the employment of random allocation.

2.4. Feasibility of a randomized, controlled trial

A feasibility study of random allocation to T380A IUD or DMPA IM contraception conducted by Family Health International (FHI 360) in four countries enrolled 368 women and concluded that such a study is feasible. Most women approached agreed to participate, more than two-thirds continued the allocated contraception method throughout the trial period, and pelvic infection rates were low23. The current ECHO study has demonstrated excellent recruitment and retention at sites in South Africa, Zambia Swaziland and Kenya despite the complexity of randomization to DMPA versus IUD versus implant. Randomization to two injectables should be considerably easier, as the great majority of women in the local settings prefer injectables.

2.5. Feasibility of offering each of the two study methods in selected study sites

The study is planned to be conducted in collaboration with the Provincial Departments of Health (Estern Cape and KwaZulu-Natal). It is the policy of the Department of Health to provide free family planning services to all women who request such services and both DMPA and NET-EN are widely available. The Department of Health has given written approval for provision of injectable contraception to women in the study.

3. Study objectives

1. To measure differences in immunological, hormonal, physiological, psychological, behavioural and hormonal effects of NET-EN versus DMPA considered to be relevant to HIV acquisition risk and method adherence.
2. To demonstrate feasibility of a proposed large randomized clinical trial to determine the relative risk of HIV acquisition.

Primary objectives

To compare immunological, hormonal, physiological psychological and behavioural effects which may impact on HIV acquisition risk among women randomly allocated to use NET-EN with those among women allocated to use DMPA.

Secondary objectives

To compare side effects and method continuation among women allocated to use NET-EN versus women allocated to use DMPA.

4. Study outcomes:

4.1 Primary outcomes

The primary hormonal outcome will be serum 17β estradiol, and the primary psychological outcome will be depression (BDI-II). The study will be powered to measure a difference of 35 pmol/L in the mean serum 17β estradiol between arms, and a difference of 2 in the mean depression scores (BDI-II). The other measures will be regarded as exploratory.

4.2 Secondary outcomes

Sentinal immune suppression markers (eg CCR5 levels on T cells and CD14+ cells from genital tract samples; plasma interferonγ).

Serum sex hormone binding globulin

Serum testosterone

Serum free testosterone

Serum progesterone

Additional depression measures

Sexual dysfunction

Sexual activity including condom use

Amenorrhoea

Weight change

Adherence

The trial will not be powered for HIV acquisition or pregnancy, but these will be measured to provide a range of plausible risk ratios and contribute data to our Cochrane review of the topic.

5. Study design

This will be a 2-arm, multicenter randomised clinical trial where one group will be allocated to NET-EN use and the other to DMPA. The group allocated to NET-EN will be the treatment group and those allocated to DMPA will be the comparison group, as the majority of women

in the research settings use DMPA. The follow up period will be a minimum of 7 months, and pending funding up to 18 months.

5.1 Methods and study procedures :

The study design, procedures, inclusion and exclusion criteria, randomization procedures, follow up and data management are outlined below.
Biological samples will be collected at baseline, 6 months and 6 months7days to measure differences between women allocated to NET-EN versus DMPA in biological parameters relevant to HIV acquisition risk. Sample size: 520 participants

5.2 Study sites

The research sites include the East London, Mdantsane, public health clinics and hospitals (Frere and Cecilia Makiwane Hopsitals), and the research site of MatCH Research Unit (MRU), University of the Witwatersrand, based in Kwazulu Natal, South Africa. These sites have been chosen as they have research and diagnostic capabilities, demonstrated by successful participation in the current ECHO study, and are research units affiliated to University of the Witwatersrand.

5.3 Study participants

Women attending health services in the collaborating Department of Health participating services and the study sites who, after routine counselling on all the methods available (Injectables, OC, IUD, Implant), request and are assessed as suitable for injectable contraception and meet the eligibility criteria, will be eligible for participation. Specific services for recruitment will include women attending family planning clinics women, women accessing pregnancy termination services and the general public.

5.3.1 Participant recruitment

Women requesting injectable contraception will be informed by the study recruitment team about the study and if they are interested , advised to go for HIV test at a health facility as the site will require recent results before they are screened for eligibility. The aim is to pre-screen all participants to reduce screen failures who might be HIV positive. Women expressing an interest will sign consent for screening and be assessed for eligibility using a screening form. A urine pregnancy test will be performed if pregnancy has not been ruled out clinically. Women who meet the entry criteria will be fully counselled and informed in their preferred language (informed consent sheet attached) and invited to participate. Emphasis will be place on HIV risk reduction including condom use. Women will be offered time to reflect and ask questions, and

to go home to consider or consult with family members if they wish to. Women who agree to participate will sign informed consent (form attached).

Exclusion of pregnancy and clinical assessment for STI's or contra-indications to family planning methods are part of the routine health services assessment for family planning and not specific to the research, and any illness or pregnancy detected will be managed in the routine service.

5.4 Eligibility Criteria

5.4.1 Inclusion criteria

- Requests injectable contraception and intends to continue contraception for at least 18 months.
- Aged 18 to 40 years. Legally competent to sign consent according to local regulations..
- Prepared to use either NET-EN or DMPA
- Prepared to accept follow-up procedures and able to fulfil these procedures, including routine HIV tests according to national guidelines (see below).
- After full counselling, decline to use PrEP
- Understands patient information form and signs informed consent.

5.4.2 Exclusion criteria

- Received DMPA in previous 6 months or NET-EN in previous 4 months
- HIV positive
- Planning to move out of study area in next 18 months
- Participating in another clinical trial
- <6 weeks postpartum or post-abortion
- Does not meet MEC criteria or local national guidelines for NET-EN or DMPA use.
- Using or intending to use medication which may interfere with biological measurements, for example steroids or drugs affecting renal function such as PrEP. Prospective participants will be fully informed about PrEP, and if interested in using PrEP, will be referred to any available local providers.Participants who, after recruitment, change their minds and decide to access PrEP services, will be kept in the study.

5.5 Sampling and allocation

The anticipated recruitment proportions will be 50% per site. Recruitment will continue at both sites until the total sample size is reached.

5.6 Sample size calculation

Primary psychological outcome: Depression

In our previous study, 24 MADRS scores in women randomized to NET-EN were 8.3 (standard deviation 7.5). Based on this standard deviation, to show a difference of 2 between the study groups with 95% certainty and 80% power would require 221 participants per group, and allowing for 15% loss to follow-up, 260 per group. (http://pharmaschool.co/size4.asp).

Primary biological outcome: serum 17β estradiol

In our previous study,[24] 17β estradiol levels in women randomized to NET-EN were 136 pmol/L (standard deviation 119). Based on this standard deviation, to show a difference of 35 pmol/L in either direction between groups with 95% certainty and 80% power would require 181 participants per group, and allowing for 15% loss to follow-up, 213 per group (http://pharmaschool.co/size4.asp).

Because of multiple outcomes to be measured, the remaining outcome comparisons will be regarded as exploratory. In general, studies of biomarkers such as immune effects have used considerably smaller sample sizes.

Feasibility of recruiting necessary sample size

Effective Care Research Unit has access to 18 primary health care centers where recruitment will take place. MatCH Research Unit has access to a large family planning clinic in Ethekwini, Kwazulu Natal.

We estimate recruitment for the 2 sites should be at least 40 per week, with completion in 12 weeks.

5.7 Description of the intervention

The administration of NET-EN or DMPA injections will be performed by an appropriately trained health professional members of the research team. If for logistical reasons an injection is given by routine health service staff, it will be documented by research staff. Clinical procedures will follow local practice guidelines. The allocated injectable method will be provided and followed up in the study site. All participants would be receiving one or other injectable method in the routine service if they were not in the trial. Both injectable methods are available in the health services, and they will be referred to the routine system for their on-going family planning care after the conclusion of the trial.

5.7.1 Drugs and devices

3. Norethisterone enanthate 200mg intramuscular (NET-EN)

4. Depot medroxyprogesterone acetate 150mg intramuscular (DMPA IM)

5.7.2 Administration of study products

DMPA administration

For women randomised to DMPA,research staff will inject the initial IM injection of DMPA (medroxyprogesterone acetate sterile aqueous suspension 150 mg per 1 mL) at enrolment. Subsequent injections will be given every 3 months at the study site.

Sites will assess participants who are more than 4 weeks late for their DMPA injections for pregnancy prior to administering a subsequent injection.

NET-EN administration

This study will use Nur-Isterat®, manufactured by Bayer HealthCare. Women randomised to NET-EN will receive the initial injection of 200 mg IM at enrolment. Subsequent injections will be given at 2-month intervals. Sites will assess participants who are more than 2 weeks late for their NET-EN injections for pregnancy prior to administering a subsequent injection.

The 2-, 4-, 8-, 10-, 14- and 16-; or 3-, 9, and 15-month visits will be contraception-only visits. Months 6, Month 6,7days, and pending funding 12 and 18 will be the study visits.

5.8 Study procedures

5.8.1 Recruitment and screening

All potentially eligible women who after routine family planning counselling have chosen an injectable progestogen, will be offered HIV testing (and CD4 counts if indicated) as part of their routine care in the health service, and using routine health service methods. HIV positive women who do not currently have routine HIV care will be referred to care within the routine health service. Women who test HIV negative will be asked if interested in participating in the trial, and assessed for inclusion.

All women being considered for recruitment will be screened for eligibility (see section 5.4). Those who are ineligible or choose not to participate will regardless be offered the contraception of their choice, according to the government family planning policy.

5.8.2 Enrolment procedures

Baseline details will be recorded from participants (see attached data collection sheet). All study procedures will be carried out in premises with appropriate privacy :

- Baseline demographic, psychological, physiological and behavioural data will be recorded
- Baseline blood (40ml venous blood) and genital tract samples (cytobrush and vaginal wall swabs) for immunological and hormonal studies, and dried blood spots for HIV PCR will be collected.
- Participants will be counselled on all HIV prevention strategies (including condom use and pre-exposure prophylaxis, and their availability in the public sector).

Table 1:Screening and Enrolment activities

Activities	Staff
Notice information +IC – [15-30min]<br>Locator infomation	Counselor
Eligibility assessment (CRF1 Scr) - [15min]<br>Demographic data	Study Nurse
HIV counselling and test – [30min]	Counselor
Clinical interview (CRF2) - [30min]	Clinician/Study Nurse

Clinical assessment (CRF3) - [30-40min]	Clinician/Study Nurse
Anthropometric measures (height,weight) (CRF3) - [10min]	Clinician/Study Nurse
BDI-II & ASEX - [15min]	Counselor
Blood and genital tract sample collection* - [30min]	Study nurse
Randomisation	Study nurse

5.8.3 Randomization procedure

Allocation lists will be prepared by SA Medical Research Council (MRC) using computer-generated random sequence in balanced blocks of variable size, stratified by study site. Women who agree to participate will be entered onto a trial register and then randomized by accessing an online randomization service hosted by SA MRC. In the event of difficulty accessing the online service, a separate series of randomized allocations will be available in sequentially numbered, sealed opaque envelopes, or by telephone back-up service. Every effort will be made to use the back-up system only when essential. The point of trial entry will be the online group allocation or opening of the envelope.

5.8.4 Follow-up visits

All participants will be asked to attend the research sites at the time of their repeat injections (2- or 3-monthly). At the 6-month and month6, 7days visit they will have blood drawn, urine and genital tract samples collected for biomarker studies and archiving. At the 6, and pending funding 12 and 18 month visits they will be offered an HIV test by study staff according to national guidelines, and a dried blood spot will be archived.

At 12 to 18 months, participants will be seen at the research site for a final dried blood spot collection and urine pregnancy test. At the end of the study, the last blood spot available will be tested for HIV by PCR. For participants with positive final HIV PCR, the baseline archived blood spots will be tested for HIV to to exclude window period infections at baseline. In the

event of failure to collect or loss of a PCR result, the relevant rapid test result will be used. In the event of discordance between PCR and rapid test results, the PCR result will be used. In the case of a positive PCR after completion of the study, the participant will be notified by the study staff.

Women who are unable to come to the research site for their final visit will have the option of an outreach visit from study personnel to perform HIV testing.

5.8.5 Study Follow-up procedures

All the participating women will be given a study calling card with a 24 hour number to call and be encouraged to report any adverse events experienced to the research team, who have the responsibility to ensure that such adverse events are appropriately managed and details of the event recorded.

In the event of a problem thought by the participant to be related to the contraceptive method, routine assessment, counselling and management will be offered according to the national contraception guidelines. Strategies to manage possible side-effects without method change will be explored. In the event of discontinuation of either method, alternative choices will be offered to participants according to national contraception guidelines.

Participants will be followed at month 6 and month 6, 7days, and pending funding 12 and 18 months. Participants will receive appropriate compensation for their time and costs for in-person visits (R250 for study visits and R100 for contraception-only visits). Every attempt will be made to contact participants who do not return for follow up including repeated calls to participant's and alternative phone numbers, and where possible home visits (provided consented to). At each contact, participants will be asked to confirm their physical address and description of where they live, as well as at least one contact number for a family member or friend that they will inform about the study and are happy to have contacted by the study team. At scheduled and unscheduled visits, the women will receive free routine clinical care and referral as needed. All participants will have access to the national HIV screening and treatment programme and STI treatment through their local clinics. If any incidental health problems are detected, the women will be referred to the relevant health facilities.

Table 2: Study follow-up activities(Every 6months)

Activities	Staff

HIV councelling and test – [30min]<br>Locator information (update)	Counselor
Clinical interview (CRF2) - [30min]	Clinician/Study Nurse
Antropometric measures (weight) (CRF3) - [10min]	Clinician/Study Nurse
Clinical assessment (CRF3) - [30min]	Clinician/Study Nurse
BDI-II & ASEX - [15min]	Counselor
Blood and genital tract sample collection* - [10min]	Study nurse

Total duration of visit not more than 2 hours.

The following data will be collected at baseline,Month 6, Month6,7days, Month 7.

- Age (at baseline only)
- Previous pregnancies, whether the pregnancy was intended or unintended, and the outcome of the pregnancy (at baseline only)
- Menstruation (heavy/mod/light/none) and symptoms eg pain
- Subjective concern about weight
- Feeling sad or depressed for no real reason
- "In the last 2 weeks have you felt unable to stop worrying, or thinking too much?"
- "In the last 2 weeks have you felt down, depressed or hopeless?"
- "In the last 2 weeks have you had thoughts and plans to harm yourself or commit suicide?"
- Current contraception, date of NET or DMPA injections (confirmed from patient-held family planning record if received outside the study site)
- Reason for discontinuation of allocated method
- Frequency of condom use (always/sometimes/never/not applicable)
- Number of sexual partners in previous 3 months

Immunological studies

The following sentinel tests are under consideration:

- CR5 levels on T cells and CD14+ cells from cytobrush sample
- plasma interferon γ

5.8.6 Contraceptive follow-up after final research visit

At the final research visit, the women will be re-counselled about their future contraceptive choices. Any who wish to switch methods will have the option to do so. Further contraceptive care will be provided within the routine provincial health services.

5.9 Criteria for discontinuation of a participant

Participants who decline to remain in the study will be discontinued. Stopping or changing the allocated method will not be a reason for discontinuation, as analysis will be by intention to treat. Pregnancy or HIV sero-conversion will not be a reason for discontinuation, as other outcomes will remain relevant.

5.10 Criteria for discontinuation of the study

An interim analysis will be performed by the statistical team and reviewed by the DSMB after 50% of women have completed the study. If the DSMB feels the data confirms significant harm or benefit of one of the study arms and recommends discontinuation of the study, enrolment will stop.

6. Study instruments

6.1 Questionnaire

A brief questionnaire will be developed to collect weight, medical and menstrual history, for comparison of other side effects as per the secondary objective (Appendix 3 case record form). This is a closed–ended questionnaire which covers all possible answers per question. The type of questions enables the generation of numeric data. The screening CRF includes eligibility check. The enrolment CRF includes medical gynaecological history, history of contraceptive use, data about HIV status, weight, and menstruation.

6.2 Beck Depression Inventory (BDI-II)

The Beck Depression Inventory is selected to evaluate depressive symptoms. It has been previously validated, used in the same cultural context and translated into the local languages IsiXhosa and IsiZulu. English, IsiZulu and IsiXhosa versions of the BDI-II will be used in this study. The BDI-II can be self administered or verbally administered by a trained administrator. In this study, verbal administration will be utilised during study follow-up interviews. The BDI-II has 21 items, and each item is rated on a four-point scale ranging from 0-3. The maximum total score is 63. According to the BDI-II manual, scores of 0-13 indicate minimal depression, scores of 14-

19 indicate mild depression, scores of 20-28 indicate moderate depression, and scores of 29-63 indicate severe depression[25] . See appendix 2 and 3 for a copy. If particpants have high scores indicationg severe depression they will be refered to a Psychologist and if unresolved referreral to a Psychiatrist will be done.

6.3 Arizona Sexual Experience Scale

The Arizona Sexual Experiences Scale (ASEX) is used to evaluate sexual functioning. This is a five-item rating scale instrument with total scores ranging from 5–30. It has been validated to be independent of the presence of a sexual partner, and can therefore be used even when study participants are not sexually active. Questions 4 and 5 of this scale are not ranked if a participant has not engaged in sexual intercourse within a week of the interview.See appendix 4 for a copy.

6.4 28 Days daily Dairy

Participating women will be asked to prospectively complete a 28-day daily diary at home of symptoms and behaviour, commencing on the day of their month 6 visit. A daily diary has been used in other studies that measure daily symptoms potentially related to the hormone fluctuations in women. Parameters measured in the daily diary include sexual interest, activity and condom use, mood, and characteristics of menstruation. To optimize compliance and the quality of self-reporting, research staff will show participants how to complete the daily diary by assisting them to complete their 'day 1' entries .

7. Project management

The Trial PIs, professional nurses and field workers who will be working as researchers will undergo a Good Clinical Practice course before they work on this trial. The site PIs will do regular checks to ensure that the field workers (trained research assistants) are practising according to the Good Clinical Practice Guidelines. Overall project management will be the responsibility of each site PI. Quality control will be monitored by external audits.

7.1 Data quality assurance

Clinical Research Forms (CRFs) will be designed and produced by the study team on site in consultation with SA MRC. CRFs will be used to collect information for statistical analysis. The study database will be constructed by SA MRC statistical services . Internal quality control will be on-site both by a second member of the research team checking forms for correct completion.

Range checks, plausibility and consistency checks will be built into the database entry system to assess consistency, accuracy and completeness of the data collected.

Guidelines will be prepared for interviewing and collecting data. If feasible, direct data entry such as REDCap will be used. Alternatively, paper CRF's will be used. The guidelines will list all data management activities and specify the roles and responsibilities of all personnel involved in the study. The local study staff will be trained in data collection, interviewing techniques and online data entry. Standard ASCII or SAS datasets will be generated from the final study database for analysis.

Study training will be carried out at each of the study sites for all members of the research team on data collection and study techniques. Specifically, participating research team members will be trained to correctly describe the study to potential participants, administer the screening and consent forms, collect contact data from participants, and correctly fill out study forms. Training will be repeated as required.

8. Data management

The SA MRC, will provide data management and statistical support for the trial. Data will be collected by research staff onto tablets or paper case record forms. The data will be entered on site onto a web based, fully GCP compliant data management system, which will be used for data entry and data validation. SA MRC will provide hosting for the system and data backups.

8.1 Confidentiality of data

Confidentiality of participant information will be assured by the following measures:

- Trial data will be entered onto CRF's with the participant's trial ID number, not name.
- Trial documents will be kept securely under lock and key in the research offices and not be accessible other than to the researchers
- Data will be entered by trial ID number onto a password-protected database to which only trial staff will have access.
- The trial report will not contain names of any participants
- After completion of the trial, the trial documents (Hard copies) will be kept for 2 years under lock and key and electronic archiving will be done for 15 years .
- Forms such as locator forms which records names and other personal indetifiers will be stored separately from the study records identified by codes.All records will be stored securely in a locked file cabinet which has limited access.

8.2 Data analysis plan

Analysis will be by intention to treat, and the results will be reported according to the CONSORT guidelines. Baseline characteristics will be compared to confirm the effectiveness of the

randomization process. Numbers and baseline characteristics of losses to follow-up will be compared between groups to detect any imbalances in loss to follow-up. Number of subjects, number of missing values and percentages will be reported for categorical variables. Number of subjects, number of missing values, minima, maxima, means and standard deviations will be reported for continuous variables. Appropriate transformation of non-normal or skewed measurement will be performed on continuous variables. If transformations do not generate a distribution approaching normality, medians and IQR will be reported.

For incidence outcomes (eg pregnancy and HIV seroconversion) with varying duration of follow up between groups, data will be adjusted for duration of follow up.

Categorical variables will be compared as risk ratios with 95% confidence intervals. For continuous data, mean differences with 95% confidence intervals will be reported.
The primary analysis will be based on all subjects with outcome data available. A sensitivity analysis will be performed that only analyzes participants who have neither discontinued nor switched contraceptive methods to assess the impact of switching and discontinuation.

8.3 Study timeline

Ethical approval of protocol: 2 months

Development of the manual of procedures: 1 month

Administrative and organizational arrangements at sites: 1 month

Training for research staff: 1 month

Recruitment: 4 months

Follow-up: 7 months, pending funding 12-18 months

Data cleaning and analysis and manuscript: 3 months

9. Main problems anticipated and proposed solutions

Loss to follow-up. Telephone calls will be used to remind participants of appointments. Mobile phone ownership is very high in our setting. We will confirm telephone numbers at each contact as well as numbers of friends or family whom participants give permission for the research team to contact. Home visits will be used if telephone contact fails, subject to specific consent for home visits having been given. If women are unable to come to the final visit in person, study personnel will go to their home or another place of their choosing, such as their local clinic, provided they have given consent for such visits.

Finally, contraceptive switching and method discontinuation may make it difficult to interpret our data. In order to minimize the impact of this, providers will carefully counsel women about side effects to expect and how to manage them.

10.Applicability of results

The rationale for this study, and the need for a more definitive answer, has been explained above. The results of this study will respond to an urgent need for better information for women and policy-makers, and will be relevant to settings similar to that of the study sites.

11. Ethical considerations:

Ethical approval will be obtained before data collection began from the Faculty of Health Sciences Human Research Ethics Committee (FHS HREC) of the University of Watwatersrand and University of Cape Town, and from the East London Hospital institutional Ethics Committee. Permission to conduct the study will be requested from the Provincial Departments of Health (state which: KZN, EC I guess WC not needed?). The South African Health Products Regulatory Authority (SAHPRA) will be notified of the study.

This study adheres to the ethical principles outlined in the Declaration of Helsinki[26] and the Constitution of the Republic of South Africa (Bill of Rights) (Devenish, 1999).

12.1. Study population, recruitment strategy and informed consent process

The inclusion criteria have been made as inclusive as possible so that a minimum number of women are excluded from the opportunity of participating in the study (see section 5.4). Research staff with GCP certification and specific training in the recruitment procedures will conduct recruitment (5.8.1). Informed consent will comply with requirements for research on human subjects.

12.2 Perceived risks and benefits of the study, both at the individual and community levels

Benefits of the study

- The study will provide sound evidence for guiding the choice of family planning methods by women in the future
- High-profile community information as part of the study will stimulate community awareness of family planning, and increase contraception uptake, leading to an overall reduction in unplanned pregnancies, pregnancy termination requests, and complications of unplanned pregnancies.
- Participants will receive high quality, respectful contraceptive care from study staff in accordance with routine health service procedures.

Risks of the study

Both methods of family planning have risks and benefits, but these are related to the participants' need for family planning, not to the research. It is not known whether one method is overall better or safer than the other. The World Health Organization recommends both methods for widespread use, and does not distinguish between their medical eligibility criteria. The discomforts of the trial procedures are completing the questionnaires and the blood and genital tract sampling. Genital tract sampling will be non-invasive, by means of cytobrush sampling from the cervix and swabs from the vaginal walls. Six-monthly HIV tests are recommended as part of the national health strategy. Knowledge of HIV results will be of benefit to participants in terms of reassurance or advice to seek appropriate care.

Adverse events will be managed according to the 'Good clinical Practice' guidelines: Any untoward occurrence will be reported as an adverse event. Any adverse event resulting in death, a life-threatening condition, hospitalisation or prolongation of hospital stay, persistent or significant disability/incapacity, or congenital anomaly, will be reported as a serious adverse event within 2 working days. Serious adverse events will be reported to the data monitoring and safety committee, who will be independent of the trial, and the relevant ethics committees.

12.3 Safeguards to protect any recognized vulnerability of the study participants

Women seeking contraceptive services may be vulnerable to coercion, especially those seeking services after a pregnancy termination. Only women who, after routine counselling in the regular services, desire a contraceptive method, and request injectable contraception, will be approached for enrolment. As detailed above (section 2.5.7), women who choose not to enrol will nonetheless be offered the contraceptive method of their choice.

12.4 Reimbursement or compensation to study participants

Participants will be reimbursed R250 for their time and transportation costs for in-person visits, and R100 for contraception only visits.

12.5 Access to treatment or counselling for conditions either identified during screening of potential participants or resulting from the study intervention

Please see sections 5.4.2 , 5.7 and 5.8.1 for details on referral processes for women who are diagnosed with HIV, pregnancy, or any other medical condition requiring treatment in the context of the study.

12.6 Responsiveness of the project to community needs and priorities

Recruitment for the ECHO study has revealed a high level of acceptance of participation among all stakeholders. Both sites have active Community Advisory Boards who have approved the study at the planning stage, and will continue to give input during the study.

12.7 Deception

Deception will not be employed in this study.

13. Gender considerations

13.1 Describe how women and men are affected by the public health need that the study addresses, and whether this is a need expressed or felt by women and/or men

Maternal deaths account for about 400 000 deaths annually, most of which take place in low-income countries. Improved information on and understanding of contraception choices will promote family planning and reduce unintended pregnancies. This research is directly related to attempts to reduce maternal mortality, as well as to identify strategies to mitigate HIV risk.

13.2 Explain how the research contributes to identifying and/or reducing inequities between women and men in sexual and reproductive health and health care.

The study will provide improved information on two confidential, woman-controlled methods for contraception. Empowering women to make their own decisions about reproduction is seen as central to promoting gender equity.

13.3 Describe measures taken to facilitate the individual participation of women or men in the research process in light of their different life situations

This project will recruit women only, and addresses gender equity by empowering women to access confidential contraception.

13.4 Describe measures taken to ensure that community involvement is inclusive

Community communication strategies will include posters informing the community at large of the study, with contact numbers for input or queries, and when possible, research staff will be interviewed on local radio, and give talks at community forums such as churches. The sites' community advisory boards will play an active part in the study planning and implementation. The great majority of researchers at all the proposed sites are female, from local communities.

14. Environmental impact of the project

As this study takes place within the context of the regular health services, the additional environmental impact is minimal. Additionally, the provision of voluntary contraceptive services may have a positive environmental impact by reducing the rate of unintended pregnancies.

15. Plans for dissemination and use of project results

Trial participants will be asked whether they wish to receive a brief report on the results of the study, and how.
The results will be important for health workers counselling women on their family planning choices, and will be made available to the Department of Health to inform family planning policy and training. Apart from primary publication in a peer-reviewed journal, the data will be incorporated into our Cochrane reviews on hormonal contraception, in the Cochrane Library, recognised as a leading source of evidence-based information.

16. Budget

Annual budget:

Injectables will be provided by routine health services as per letter from the Deputy Director-General, Dept of Health

Data management, statistical analysis and trial monitoring will be covered by the SA MRC.

Certain laboratory studies and sample archiving will be covered by Department of Molecular and Cell Biology, University of Cape Town (Prof Janet Hapgood)
Remaining budget (Trial staff, management, supplies, sample transport, laboratory tests, participant costs, etc.): 2017/18 financial year: R4 967 770 and 2018/19 financial year: R4 967 77.

Total Budget : R 9 935 540

Appendix 1: 28 Days Daily Diary

WHICH INJECTION STUDY (PART 1)

Identification

Subject number

Date of birth D D M M M Y Y

Start date D D M M M Y Y

End date D D M M M Y Y

Please tell us what happened from mid-day yesterday until mid-day today?

	1	2	3	4	5	6	7	8	9	10	11	12	13	14	15	16	17	18	19	20	21	22
**1 Have you been menstruating?** 0=No 2.Light 1.Normal 3.Heavy 1																						
**2 Has your menstruation been?** 0.No menstruation 2.Mild pain 1.Painless menstruatior 3.Severe pain 2																						
**3 Have you had sexual intercourse?** 0.No intercourse if yes 2.Casual partner 1.Steady partener only 3																						
**4 If yes Q4 sexual intercourse? Was condom used?** 0.No intercourse 2.No condom 1.Yes condom 4																						
**5 Have you felt sad for no real reason ?** 0 = No 1 = Yes 5																						
**6 Have you felt an urge to have sexual intercourse?** 0 = No 1 = Yes 6																						
**7 Did you feel that your partner loves you?** 0 .No 2 =No partner 1 .Yes 7																						

Daily Diary

17 .References

[1] Farley TM, Lusti-Narasimhan M. Hormonal contraception and risk of HIV acquisition: a difficult policy position in spite of incomplete evidence. Reprod Health Matters 2012 Dec;20(39 Suppl):14-7

[2] Rodriguez, M.I., Reeves, M.F. and Caughey, A.B., 2012. Evaluating the competing risks of HIV acquisition and maternal mortality in Africa: a decision analysis. BJOG: An International Journal of Obstetrics & Gynaecology, 119(9), pp.1067-1073.

[3] Thomson KA, Hughes J, Baeten JM, John-Stewart G, Celum C, Cohen CR, Ngure K, Kiarie J, Mugo N, Heffron R. Increased Risk of Female HIV-1 Acquisition Throughout Pregnancy and Postpartum: A Prospective Per-coital Act Analysis Among Women with HIV-1 Infected Partners. J Infect Dis. 2018 Mar 5. doi: 10.1093

[4] Joseph Davey D, Farley E, Gomba Y, Coates T, Myer L. Sexual risk during pregnancy and postpartum periods among HIV-infected and -uninfected South African women: Implications for primary and secondary HIV prevention interventions. PLoS One. 2018 Mar 6;13(3):e0192982. doi: 10.1371

[5] Singata-Madliki M, Hofmeyr GJ, Lawrie TA. The effect of depot medroxyprogesterone acetate on postnatal depression: a randomised controlled trial. J Fam Plann Reprod Health Care 2016 Mar 30. pii: jfprhc-2015-101334. doi: 10.1136/jfprhc-2015-101334

[6] Hofmeyr GJ, Singata M, Lawrie TA, Temmerman M. Interpretation, communication, and mechanisms of associations between injectable contraception and HIV risk. Lancet HIV. 2015 Sep;2(9):e365. doi: 10.1016/S2352-3018(15)00153-8. Epub 2015 Aug 26

[7] Draper BH, Morroni C, Hoffman M, Smit J, Beksinska M, Hapgood J, et al. Depot medroxyprogesterone versus norethisterone oenanthate for long-acting progestogenic contraception. Cochrane Database Syst Rev 2006;(3):CD005214.

[8] Department of Health Republic of SA. Contraceptive Clinical Guidelines. Pretoria, South Africa: Department of Health, Republic of South Africa; 2012.

[9] Shisana O, Simbayi LC, Rehle T, Zungu NP, Zuma K, Ngogo N, et al. South African National HIV Prevalence, Incidence, Behaviour and Communication Survey, 2008: The Health of Our Children. HSRC Press; 2010.

[10] Noguchi LM, Richardson BA, Baeten JM, Hillier SL, Balkus JE, Chirenje ZM, Bunge K, Ramjee G, Nair G, Palanee-Phillips T, Selepe P, van der Straten A, Parikh UM, Gomez K, Piper JM, Watts DH, Marrazzo JM; VOICE Study Team. Risk of HIV-1 acquisition among women who use diff erent types of injectable progestin contraception in South Africa: a prospective cohort study. Lancet HIV. 2015 Jul;2(7):e279-87.

[11] Hild-Petito S, Veazey RS, Larner JM, Reel JR, Blye RP. Effects of two progestin-only contraceptives, Depo-Provera and Norplant-II, on the vaginal epithelium of rhesus monkeys. AIDS Res Hum Retroviruses 1998 Apr;14 Suppl 1:S125-S130

[12] Ildgruben AK, Sjoberg IM, Hammarstrom ML. Influence of hormonal contraceptives on the immune cells and thickness of human vaginal epithelium. Obstet Gynecol 2003 Sep;102(3):571-82

[13] Bahamondes L, Trevisan M, Andrade L, Marchi NM, Castro S, Diaz J, et al. The effect upon the human vaginal histology of the long-term use of the injectable contraceptive Depo-Provera. Contraception 2000 Jul;62(1):23-7.

[14] Miller L, Patton DL, Meier A, Thwin SS, Hooton TM, Eschenbach DA. Depomedroxyprogesterone-induced hypoestrogenism and changes in vaginal flora and epithelium. Obstet Gynecol 2000 Sep;96(3):431-9.

[15] Wieser F, Hosmann J, Tschugguel W, Czerwenka K, Sedivy R, Huber JC. Progesterone increases the number of Langerhans cells in human vaginal epithelium. Fertil Steril 2001 Jun;75(6):1234-5

[16] Mauck CK, Callahan MM, Baker J, Arbogast K, Veazey R, Stock R, et al. The effect of one injection of Depo-Provera on the human vaginal epithelium and cervical ectopy. Contraception 1999 Jul;60(1):15-24

[17] Sheffield JS, Wendel GD, Jr., McIntire DD, Norgard MV. The effect of progesterone levels and pregnancy on HIV-1 coreceptor expression. Reprod Sci 2009 Jan;16(1):20-31.

[18] Cherpes TL, Busch JL, Sheridan BS, Harvey SA, Hendricks RL. Medroxyprogesterone acetate inhibits CD8+ T cell viral-specific effector function and induces herpes simplex virus type 1 reactivation. J Immunol 2008 Jul 15;181(2):969-75.

[19] Bamberger CM, Else T, Bamberger AM, Beil FU, Schulte HM. Dissociative glucocorticoid activity of medroxyprogesterone acetate in normal human lymphocytes. J Clin Endocrinol Metab 1999 Nov;84(11):4055-61.

[20] Trunova N, Tsai L, Tung S, Schneider E, Harouse J, Gettie A, et al. Progestin-based contraceptive suppresses cellular immune responses in SHIV-infected rhesus macaques. Virology 2006 Aug 15;352(1):169-77

[21] Hel Z. Personal communication. 2013

[22] Quispe Calla NE, Vicetti Miguel RD, Boyaka PN, Hall-Stoodley L, Kaur B, Trout W, Pavelko SD, Cherpes TL.Medroxyprogesterone acetate and levonorgestrel increase genital mucosal permeability and enhance susceptibility to genital herpes simplex virus type 2 infection. Mucosal Immunol. 2016 Nov;9(6):1571-1583

[23] Feldblum PJ, Caraway J, Bahamondes L, El-Shafei M, Quan HD, Morales E, et al. Randomized assignment to copper IUD or depot-medroxyprogesterone acetate: feasibility of enrollment, continuation and disease ascertainment. Contraception 2005 Sep;72(3):187-91.

[24] Lawrie TA, Hofmeyr GJ, De Jager M, Berk M, Paiker J, Viljoen E. A Double blind randomised placebo controlled trial of postnatal norethisterone enantate: the effect on postnatal depression and serum hormones. Br J Obstet Gynecol 1998; 105: 1082-1090.

[25] Beck, A. T., Ward, C., & Mendelson, M. (1961). Beck depression inventory (BDI). Arch Gen Psychiatry, 4(6), 561-571.

[26] World Medical Association, 2015. WMA Declaration of Helsinki—ethical principles for medical research involving human subjects. 2013. Google Scholar.
