# Supplementary material for: Effects of injectable contraception with depot medroxyprogesterone acetate or norethisterone enanthate on estradiol levels and menstrual, psychological and behavioral measures relevant to HIV risk: The WHICH randomized trial
Source: PLoS One. 2024 Mar 26;19(3):e0295764. doi: 10.1371/journal.pone.0295764 (PMC10965066; doi:10.1371/journal.pone.0295764)
Supplement: Attachment — Submitted filename: Response to reviewers.docx [file pone.0295764.s005.docx]

Reference: PONE-D-23-10439

Title: Effects of injectable contraception with depot medroxyprogesterone acetate or norethisterone enanthate on estradiol levels and menstrual, psychological and behavioral measures relevant to HIV risk: the WHICH randomized trial

**Responses to editor’s and reviewers’ comments**

| **Comments** | **Author responses** |
| --- | --- |
| **Academic Editor** | |
| (a) How was randomization concealed to ensure unpredictability of the subsequent arm assignment by those randomizing participants? | We have added the following sentence to the manuscript: “Those enrolling participants could not predict the randomization sequence.” (Page 10)  Additional details that were in the manuscript to explain the randomization process were as follows under the ‘**Randomization and masking’** section: “Allocation lists were prepared independently by SA Medical Research Council (MRC) using computer-generated random sequence in balanced blocks of variable size, stratified by study site. Participants who agreed to participate were entered onto a trial register and then randomized by accessing the online randomization REDCap programme [37]. In the event of difficulty accessing the online service, a separate series of randomized allocations was available in sequentially numbered, sealed opaque envelopes, or by telephone back-up service.” (Page 10) |
| (b) What is the usefulness of p-value in table 1 for a randomized trial?, using the coinvention of p . 0.05. | We agree p-values are inappropriate for baseline values and have deleted them from Table 1. (Pages 16-17) |
| (c) Why >80% were unemployed? Was this systematic or came out of a random process? | We have added the following text (added text in bold) to the manuscript to explain this finding: “Most participants were unemployed, **which is typical of the low-income populations we serve.**” (Page 16) |
| **Journal Requirements** | |
| 1. Please ensure that your manuscript meets PLOS ONE's style requirements, including those for file naming | This has been done. |
| 2. We note that you have indicated that data from this study are available upon request. PLOS only allows data to be available upon request if there are legal or ethical restrictions on sharing data publicly. For information on unacceptable data access restrictions, please see [http://journals.plos.org/plosone/s/data-availability#loc-unacceptable-data-access-restrictions](https://protect-za.mimecast.com/s/q_qMC76y5XtAQzg73I08YmT).  In your revised cover letter, please address the following prompts:  a) If there are ethical or legal restrictions on sharing a de-identified data set, please explain them in detail (e.g., data contain potentially identifying or sensitive patient information) and who has imposed them (e.g., an ethics committee). Please also provide contact information for a data access committee, ethics committee, or other institutional body to which data requests may be sent.  b) If there are no restrictions, please upload the minimal anonymized data set necessary to replicate your study findings as either Supporting Information files or to a stable, public repository and provide us with the relevant URLs, DOIs, or accession numbers. Please see http://www.bmj.com/content/340/bmj.c181.long for guidelines on how to de-identify and prepare clinical data for publication. For a list of acceptable repositories, please see http://journals.plos.org/plosone/s/data-availability#loc-recommended-repositories.  We will update your Data Availability statement on your behalf to reflect the information you provide. | Thank you for this comment. We do not have ethical or legal restrictions on sharing a de-identified data set publicly. Hence, we will upload the data set onto a portal site of South African Medical Research Council portal upon acceptance of the paper. The link will only be assessable upon acceptance of publication. We have added the following sentence under “Data availability”:  “The de-identified dataset is available on the South African Medical Research Council portal via the following link …“(link to be added upon acceptance) (Page 30) |
| 3. We note that the original protocol that you have uploaded as a Supporting Information file contains an institutional logo. As this logo is likely copyrighted, we ask that you please remove it from this file and upload an updated version upon resubmission. | Thank you for noting this. The logo has been removed. |
| **Reviewers’ comments** | |
| 1. Is the manuscript technically sound, and do the data support the conclusions? Reviewer #1: Yes Reviewer #2: Yes | Thank you |
| 2. Has the statistical analysis been performed appropriately and rigorously?  Reviewer #1: Yes Reviewer #2: Yes | Thank you |
| 3. Have the authors made all data underlying the findings in their manuscript fully available? Reviewer #1: Yes Reviewer #2: Yes | Thank you |
| 4. Is the manuscript presented in an intelligible fashion and written in standard English? Reviewer #1: Yes Reviewer #2: Yes | Thank you |
| **Reviewer#1** | |
| Reviewer #1: A two-arm randomized clinical trial was conducted to compare hormonal, behavioral, and menstrual effects between the two injectable contraceptives. At 25 weeks, estradiol levels were statistically significantly lower than at baseline for both contraceptives. No statistical difference was observed between the two methods. Behavioral outcomes suggest less sexual exposure with NET-EN than DMPA-IM. | This is noted. |
| Minor revisions:  1- Lines 277-288: State the statistical testing method which achieves 80% power. Perhaps the power estimates are based on Student’s t-tests. | Thank you for this comment. We have added the statistical testing method of a Student’s t-test with assumed common standard deviation to the statistical analysis section: “Using a two-sample Student’s t-test and assuming a common SD of 119 pmol/L, a sample size of 181 participants per group was required to show a difference of 35 pmol/L in either direction with 95% certainty and 80% power.” (Page 13) |
| 2- Line 302: Indicate if the interaction effect of arm and time point was included. The results presented in Table 2 should be based on a test of the interaction effect. If the interaction effect is significant, provide an interpretation of the results, but do not test main effects because the tests for main effects are uninteresting in light of significant interactions. If the interaction effects are non-significant, drop the interaction effects from the model and test the main effects. Determining which results to present when testing interactions is often a multi-step process. | Thank you for these suggestions. The interaction effect of arm and time was not significant (p = 0.866). When dropping the interaction effect and testing the main effect, there is no significant difference between arms (p = 0.556), but a significant difference between time points (p < 0.001). Hence, we have opted to present results for each time point, as well as considering the change from baseline for each participant. We believe this presentation will be clinically relevant.  We have added the following text to the manuscript:  “Since the interaction effect between arm and time point were not significant (p = 0.866), we present the tests for the main effects in Table 2.” (Page 17) |
| 3- In the statistical analysis section, state and describe the use of mixed-effects linear regression. Also state the underlying covariance structure used and the criteria for selecting it. | Thank you for this comment. We agree. We have added the following text:  “Median serum estradiol was compared across arms and across time points using a mixed-effects linear regression with random effects for site and participant ‘and a diagonal covariance structure (selected on the assumption that levels within site and participant are not correlated). Through the incorporation of random effects, the mixed-effects linear regression model is able to account for the hierarchical structure of the data with participants being clustered by site and the repeated estradiol measurements clustered within participants.” (Pages 14-15) |
| 4- Tables 5-6: Provide a more descriptive column label than “Stat.” | Thank you for noting this. We have replaced “Stat” with “**Results**^a^” which we clarify in a footnote below the tables.  “^a^Results expressed as n-value (percent), mean (standard deviation) or median (interquartile range).” (Pages 21 and 23) |
| 5- Cite the statistical software used for the analysis. | We have added a citation for the statistical software used for the analysis: “All analysis was conducted using Stata version 16 (StataCorp. 2019. Stata Statistical Software: Release 16. College Station, TX: StataCorp LLC).” (Page 15) |
| **Reviewer #2** | |
| Reviewer #2: This paper is based on a open-label trial that provides valuable contributions to this area of research. This paper would benefit from clarifying the exposures, outcomes and aims of the paper. My understanding was the aim of the paper was to look at the impacts of DMPA-IM and NET-EN on hormonal (estradiol levels measured via immunoassay), psychological (depression score, via verbal administration), behavioural (Arizona Sexual Experiences Scale, questionnaire daily), and menstrual (structured questionnaire & daily diary) factors. Whilst this is what seems to be the aim, the results present much more broad results than this which makes it slightly confusing and feels like there is too much information trying to be displayed in one paper. For example I am not sure what aim table 3 or table 4 is supposed to be addressing | Thank you for this comment, and for highlighting that we had not specified that one of our aims was to assess whether the physiological and behavioral outcomes were associated with estradiol levels (results shown in Tables 3 and 4). We have added this aim to the “**Aim, design and setting”** section (page 8), as well as to the **abstract** (page 3). The following text has been added to the abstract, “We aimed in this paper to investigate the effects of DMPA-IM and NET-EN on estradiol levels, measures of depression and sexual activity and menstrual effects, relevant to HIV risk; and to ascertain whether these measures are associated with estradiol levels” (Page 3), and to the manuscript: “and whether these measures are associated with estradiol levels” (Page 8)  We have also included a sentence in the abstract on the general results of these associations from Tables 3 and 4: “We did not find a clear association between estradiol levels and sexual behavior, depression and menstrual effects” (Page 4).  In addition, we have defined the primary and secondary aims of the WHICH trial more clearly in the “**Aim, design and setting”** section (page 8). The following has been added: “The primary aim of the WHICH study was to investigate the effects of DMPA-IM and NET-EN on estradiol levels and depression. Secondary aims included other hormonal effects, sexual behavioral, menstrual and immune effects within and between the two products. In this paper we report on the primary outcomes as well as some of the secondary outcomes, namely sexual behavioral and menstrual effects; and whether these measures are associated with estradiol levels.” (Page 8) |
| The diaries are a really interesting aspect of this study but I feel like they get brushed over without much discussion - as said earlier it feels like too much data is trying to be reported in this paper and would be better if it focussed more specifically on certain data as opposed to trying to cover it all. For table 6, the data is quite confusing and it isn't intuitive what to take away from the table. | Thank you for this comment. We used the daily diary approach as a novel strategy to overcome limitations of data based on recall. We have included all the data in the tables as we believe it is important to avoid selective data reporting. However, we have highlighted important findings in the text under the Results and Discussion sections.  In addition, we have added some text in the results section to clarify the usefulness of the daily diary: “To obtain more information about psychological behavior relevant to depression, sexual behavior and menstrual effects, in addition to the data in Table 5 for baseline and at 25 weeks, a 28-day daily diary was initiated at 24 weeks. This novel strategy was used to overcome potential limitations of data based on recall. The effects of DMPA-IM and NET-EN on depression, sexual behavior and menstrual effects were assessed from the combined data in Tables 5 and 6” (Page 22)  We also clarify some differences between tables 5 and 6 (pages 19 and 22).  The take-away message from Tables 5 and 6 all relate to sexual behavior, depression and menstrual effects, the main findings on which are now reported in the first paragraph of the discussion (Page 24). More detail was and still is given under specific paragraphs on these measures, in the Discussion and Results sections. |
| The paper mentions the 'significance level is <0.05', it would be good to explain the justification for this. Within epidemiology we are trying to shift away from this as an arbitrary cut off. Also moving away from data being deemed 'statistically significant' but focusing more on the strength of evidence. | Thank you. We agree that the ‘all-or-nothing’ dichotomous approach to research is being questioned in favor of a more holistic evaluation of the strength of evidence. We have also presented the effect sizes, precision estimates, and commented on the biological plausibility and consistency of the results with other studies. The significance level serves as a guideline of the results presented and does not solely account for our conclusions.  We have added the following text: “The strength of this evidence is high due to the randomized study design and the consistency of results across the outcomes measured” to the abstract results (page 4) and to the Discussion (page 24). |
| On page 16 in the final paragraph it states 'The level (of estradiol) at 25 weeks was 9% lower with NET-EN than DMPA-IM', but then a few sentences later it states 'Median estradiol decreased by 14.8 pmol/L more in the DMPA arm than the NET-EN arm, a difference which was not statistically significant (p = 0.467)'. These two statements seem contradictory? | The apparent contradiction is a function of the non-normal distribution of the data. We have re-stated the following to avoid confusion: “The median decreases were not statistically different between the groups (p = 0.467).” (Pages 17-18) |
| Table 2 - please check the numbers, the median change from baseline don't seem to add up. | Thank you for this comment. To clarify, the median change from baseline presented in Table 2 is not the difference between the baseline and 25 week medians. The change from baseline was calculated for each participant and the median of those changes is reported. |
| I would suggest rewording the discussion so the key findings of this paper come first. | Thank you for this comment. We agree. We have added the following text to the first paragraph of the discussion: “We found a substantial and similar reduction in estradiol levels to postmenopausal levels with DMPA-IM and NET-EN. Compared with DMPA-IM, NET-EN users reported significantly less amenorrhoea, less sexual activity, more condom use, and more feeling sad and unloved. These behavioral outcomes suggest less sexual exposure with NET-EN. The strength of this evidence is high due to the randomized study design and the consistency of results across the outcomes measured.” (Page 24) |
